# Transcriptional profiles of *Microcystis* reveal gene expression shifts that promote bloom persistence in *in situ* mesocosms

**DOI:** 10.1128/spectrum.01369-24

**Published:** 2024-11-18

**Authors:** Lauren E. Krausfeldt, Paisley S. Samuel, Robert P. Smith, Hidetoshi Urakawa, Barry H. Rosen, Rita R. Colwell, Jose V. Lopez

**Affiliations:** 1 Department of Biological Sciences, Guy Harvey Oceanographic Center, Nova Southeastern University, Dania Beach, Florida, USA; 2 Cell Therapy Institute, Kiran Patel College of Allopathic Medicine, Nova Southeastern University, Fort Lauderdale, Florida, USA; 3 Department of Medical Education, Kiran Patel College of Allopathic Medicine, Nova Southeastern University, Fort Lauderdale, Florida, USA; 4 Department of Ecology and Environmental Studies, Florida Gulf Coast University, Fort Myers, Florida, USA; 5 Institute for Advanced Computer Studies, University of Maryland College Park, College Park, Maryland, USA; Connecticut Agricultural Experiment Station, New Haven, Connecticut, USA

**Keywords:** microcystin, *Microcystis*, harmful algal bloom, transcriptomics, cyanophages, toxin-antitoxin systems, Lake Okeechobee, Caloosahatchee River, phage, algae, cyanobacteria

## Abstract

Harmful algal blooms caused by cyanobacteria threaten aquatic ecosystems, the economy, and human health. Previous work has tried to identify the mechanisms that allow blooms to form, focusing on the role of nutrients. However, little is known about how introduced nutrients influence gene expression *in situ*. To address this knowledge gap, we used *in situ* mesocosms initiated with water experiencing a *Microcystis* bloom. We added pulses of nutrients that are commonly associated with anthropogenic sources to the mesocosms for 72 hours and collected samples for metatranscriptomics to examine how the physiological function of *Microcystis* and bloom status changed. The addition of nitrogen (N) as urea, but not the addition of PO_4_, resulted in conspicuous bloom persistence for at least 9 days after the final introduction of nutrients. The addition of urea initially resulted in the upregulation of photosynthesis machinery, as well as phosphate, carbon, and N transport and metabolism. Once *Microcystis* presumably became N-replete, upregulation of amino acid metabolism, microcystin biosynthesis, and other processes associated with biomass generation occurred. These capacities coincided with the upregulation of toxin-antitoxin systems, CRISPR-*cas* genes, and transposases suggesting that phage defense and genome rearrangement are critical in bloom persistence. Overall, our results show the stepwise transcriptional response of a *Microcystis* bloom to the introduction of nutrients, specifically urea, as it is sustained in a natural setting. The transcriptomic shifts observed herein may serve as markers of the longevity of blooms while providing insight into why *Microcystis* blooms over other cyanobacteria.

**IMPORTANCE:**

Harmful algal blooms represent a threat to human health and ecosystems. Understanding why blooms persist may help us develop warning indicators of bloom persistence and create novel mitigation strategies. Using mesocosm experiments initiated with water with an active bloom, we measured the stepwise transcription changes of the toxin-producing cyanobacterium *Microcystis* in response to the addition of nutrients that are important in causing blooms. We found that nitrogen (N), but not phosphorus, promoted bloom longevity. The initial introduction of N resulted in the upregulation of genes involved in photosynthesis and N import. At later times in the bloom, upregulation of genes involved in biomass generation, phage protection, genomic rearrangement, and toxin production was observed. Our results suggest that *Microcystis* first fulfills nutritional requirements before investing energy in pathways associated with growth and protection against competitors, which allowed bloom persistence more than a week after the final addition of nutrients.

## INTRODUCTION

Cyanobacterial harmful algal blooms (cyanoHABs) are a major concern in freshwater ecosystems worldwide (1). CyanoHABs have significant negative consequences on the health of humans (2), animals (3), ecosystems (4), and the economy (5–7). For example, microcystin, a cyanotoxin produced by common bloom-forming cyanobacteria like *Microcystis*, can contaminate drinking water (8). Ingestion of these toxins can lead to liver damage, gastrointestinal distress, and an increased risk of cancer (9). Blooms also create hypoxic conditions, produce taste, odor, and estrogenic compounds, and shade benthic macrofauna (1, 10, 11). Climate change is expected to increase the occurrence and expand the geographical range of cyanoHABs (1, 12). Thus, new approaches toward predicting, preventing, and disrupting the formation of cyanoHABs are needed (13, 14).

Anthropogenic activities have long been known to impact the formation and severity of cyanoHABs. Runoff from agricultural, farming, and urban environments increases the abundance of nutrients driving cyanoHABs, chiefly nitrogen (N) and phosphorus (P) in freshwater ecosystems (15–19). In addition to their abundance, nutrient bioavailability and ratios of nutrients impact cyanoHABs. Much of the previous work that identified these relationships focused on laboratory experiments using cultures of cyanobacteria. Although these experiments revealed important effects of nutrients on cyanobacterial growth and toxin production, they do not necessarily represent bloom dynamics in the natural environment. In nature, many interacting chemical and physical factors influence bloom proliferation or microcystin production (20–22). Furthermore, natural bloom populations comprise many bacterial and eukaryotic species competing for nutrients (23), with most of the bacterial species uncultivable. Thus, addressing every interacting factor that alters bloom dynamics in the laboratory is nearly impossible and cannot be directly translated to the environment.

*In situ* mesocosm experiments offer an alternative to laboratory experiments for studying how nutrients impact blooms. Importantly, mesocosms can be more representative of natural bloom dynamics. Studies that have previously utilized mesocosms generally track the influence of nutrients by measuring growth, cyanobacterial composition, and microcystin concentration (24–26). However, they do not provide insight into the underlying physiology or the systems biology of *Microcystis* and the accompanying community driving the maintenance or proliferation of a bloom.

One approach to understanding bloom dynamics is metatranscriptomics, which allows the examination of transcripts from uncultivated species. This approach has been applied to freshwater blooms (27) and has allowed us to understand changes in seasonal (28) and diel (29) gene expression during a bloom, the dynamics leading to degradation of microcystin (30), and the effect of bloom mitigation strategies on gene expression (31). They have also revealed interactions between cyanobacteria and phage during a bloom (32). However, how changes in community composition and gene expression in response to anthropogenically introduced nutrients occur *in situ* have yet to be elucidated. Revealing changes in gene expression may lead to the identification of functions that influence bloom duration or intensification.

To address this, we performed *in situ* mesocosms on the Caloosahatchee River (CR), southwest Florida, during a *Microcystis* bloom. The CR is a major waterway in southwest Florida (USA) that serves as a freshwater-marine continuum (33). It receives outflows from Lake Okeechobee (Lake O), which is eutrophic and experiences annual cyanoHABs caused by *Microcystis*. Lake O is the largest lake (1,720 km^2^) in the southeastern United States. Controlled water releases into the CR, which flows west toward the Gulf of Mexico, and the St. Lucie River, which flows east toward the Atlantic Ocean (34, 35), are needed to protect the integrity of the earthen dike surrounding it. In the last three decades, these water releases have been speculated to cause the massive accumulations of *Microcystis* biomass in the Caloosahatchee and St. Lucie Rivers (36–39). Accordingly, studying cyanoHAB dynamics in Lake O is critical for preserving the CR.

## MATERIALS AND METHODS

### Mesocosm setup and experimental design

*In situ* mesocosm experiments were carried out on the CR at the Franklin S-79 Lock and Dam in Fort Myers (Florida, USA). In this area, water flow is significantly reduced due to the lock system. Twelve fiberglass chambers (0.75 m [2.5 ft] diameter × 1.5 m [5 ft] deep), closed at the bottom but open at the surface, were arranged in groups of four in three floating metal chambers (Fig. S1). Water (556 L) was collected from the CR at the Franklin Lock and was pumped into each chamber from the shore. Two sets of three mesocosm chambers were for nutrient addition experiments; one set of three served as a no-nutrient addition control. Nutrient was added at time (T, hours) 0, T24, and T48 as 98.5% dibasic dodecahydrate sodium phosphate (Na_2_HPO_4_·12H_2_O) and urea (CH_4_N_2_O, Table S1). Results from the fourth set of three chambers will not be described in this study. Concentrations of nutrient were chosen to simulate a high nutrient loading event and create favorable conditions for a bloom. To do this, nutrients needed to be above background concentrations of N and P. To determine environmentally relevant concentrations, TN, NO_3_, NH_4_, and PO_4_ data from DBHydro, SFWMD’s environmental database that stores historic and current water quality data (https://www.sfwmd.gov/science-data/dbhydro), were analyzed from previous years (January 2015 to April 2020). Data specific for organic N and bioavailable N were not available. As nutrient concentration varies with season, flow, and water releases from Lake O, the maximum concentration of TN, NO_3_+NH_4_, and PO_4_ was considered as a baseline for background. The maximum average monthly concentration observed for TN was 132 µmol N, for NO_3_+NH_4_ was 40 µmol N, and for PO_4_ was 6.8 µmol P. By incrementally increasing the molar concentrations of N and P in the mesocosm experiments, environmentally relevant concentrations were captured while increasing the probability of raising concentrations above background.

### Sample collection

Surface water (5-cm depth) samples from the mesocosm and CR were collected using 1 L high-density polyethylene bottles. Prior to collection, each bottle was decontaminated by washing with hot water and Liquinox soap, rinsing with tap water three times, rinsing with 10% wt/wt hydrochloric acid, and rinsing with analyte-free water three times. Samples for nucleic acid extraction and nutrient measurements were collected before the addition of nutrient at T0, T24, T48, and T72. Additionally, samples for nutrient measurements were collected approximately 60 minutes after the addition of nutrients to allow ample time for mixing (Table S2). In addition to the collection of water from mesocosms, surface water was collected daily from the CR to serve as ambient controls (Table S2). Samples for nucleic acid extraction from the CR and the mesocosms (100–200 mL) were filtered through 0.22-µm Sterivex-GP pressure filter cartridges on-site as quickly as possible after sample collection. The Sterivex cartridges were immediately flash frozen using a dry ice/ethanol bath and stored at −80°C for further analysis. For nitrate and nitrite (NO_3_+NO_2_), ammonium (NH_4_), phosphate (PO_4_), and urea measurements, a 50 mL aliquot of water was filtered through a 0.7-µm GF/F filter and kept on ice until they could be frozen at −20°C in the laboratory later that same day. Aliquots of 50 mL of whole water were preserved at 4°C for total N (TN) and total P (TP) measurements. NO_3_+NO_2_, NH_4_, and PO_4_ were measured at the certified water testing laboratory, CAChE Nutrient Analysis Core Facility, at Florida International University. Urea was measured using the Urea Nitrogen (BUN) Colorimetric Detection Kit (Invitrogen, Carlsbad, CA, USA) as recommended by the manufacturer. The assay was measured using a Victor X4 microplate reader (Perkin Elmer, Waltham, MA, USA). The South Florida Water Management District (SFWMD) also collected surface water samples from Lake O (5-cm depth) monthly in 2020 and 2021 (Table S3) as described by Krausfeldt et al. (40).

### Measurement of water quality parameters and nutrients

Temperature, conductivity, pH, turbidity, dissolved oxygen (DO), fluorescent dissolved organic matter (fDOM), phycocyanin (PC), and chlorophyll a (chl *a*) were measured at T0, T24, T48, T72, and T264 in the river and the mesocosm chambers. Measurements were taken before nutrient addition using a YSI EXO multiparameter water quality sonde. Microcystin concentration was determined using an enzyme-linked immunosorbent assay (Beacon Analytic Systems Inc., Maine, USA) as described previously (41). For measuring chl *a* in the river, extraction was performed using 90% acetone and was quantified using a Trilogy Laboratory Fluorometer (Turner Designs, San Jose, CA, USA) as reported previously (41). Algal colony abundance was quantified by filtering 50 mL of water through a GF/F filter and was counted using a dissection microscope.

### Nucleic acid extraction

Before nucleic acid extraction, all tools and equipment were decontaminated with RNase Away (Thermo Scientific, 21-236-21). To extract nucleic acids, Sterivex cartridges were first opened using a pipe cutter, and the filter was removed with a scalpel. DNA and RNA were extracted with the ZymoBiomics DNA/RNA Miniprep kit in a laminar flow hood. The manufacturer’s protocol was followed to extract DNA and RNA in parallel, except the DNase treatment was performed twice sequentially before RNA was purified. DNA and RNA samples were sent to CosmosID for subsequent library preparation and sequencing. Sterivex cartridges from Lake O were extracted for total DNA using the DNeasy Powerlyzer PowerSoil DNA extraction kit (Qiagen, 12855-100) following the manufacturer’s protocol as described by Krausfeldt et al. (40).

### CosmosID library construction

DNA libraries were prepared from DNA extracts from the river and Lake O in 2020 and 2021 using the Nextera XT DNA Library Preparation Kit (Illumina) and IDT Unique Dual Indexes with a total DNA input of 1 ng. Genomic DNA was fragmented using a proportional amount of Illumina Nextera XT fragmentation enzyme. Unique dual indexes were added to each sample followed by 12 cycles of PCR to construct libraries. DNA libraries were purified using AMPure magnetic beads (Beckman Coulter) and were eluted in EB buffer (Qiagen). DNA libraries were quantified using Qubit4 fluorometer and Qubit dsDNA HS Assay Kit. Libraries were then sequenced on an Illumina HiSeq X platform 2 × 150 bp.

Isolated RNA from the mesocosms and CR samples was assessed for quality by a High Sensitivity RNA TapeStation (Agilent Technologies Inc., California, USA) and was quantified using a Qubit 2.0 RNA HS assay (Thermo Fisher, Massachusetts, USA). Ribosomal RNA depletion was performed with QIAseq FastSelect HMR and 5S/16S/23S kit (Qiagen, Hilden, Germany) per the manufacturer’s instructions. RNA libraries were constructed according to NEBNext UltraTM II Directional RNA Library Prep Kit for Illumina (New England BioLabs Inc., Massachusetts, USA). The quantity of the final RNA library was assessed using a Qubit 2.0 (Thermo Fisher, Massachusetts, USA). The quality was assessed using a TapeStation HSD1000 ScreenTape (Agilent Technologies Inc., California, USA). The final library size was approximately 350 bp with an insert size of approximately 200 bp. Illumina 8-nt unique dual-indices were used. Equimolar pooling of libraries was performed and sequenced on an Illumina Novaseq S4 (Illumina, California, USA) with a read length configuration of 150 PE for 60M PE reads per sample.

### Taxonomic analysis and functional analysis

Reads from metagenomes and metatranscriptomes were annotated taxonomically and functionally using the CosmosID-HUB as described by Krausfeldt et al. (40). Briefly, for taxonomic ID, the system utilizes a high-performance data-mining k-mer algorithm that disambiguates millions of short sequence reads into the discrete genomes engendering the particular sequences. Relative abundances of results of taxa were used for analysis. For functional analysis, metagenomic reads were trimmed and filtered for quality and then subjected to a translated search against a comprehensive and non-redundant protein sequence database, UniRef90 (42). The mapping of metagenomic reads to gene sequences is weighted by mapping quality, coverage, and gene sequence length to estimate community-wide weighted gene family abundances as described by Franzosa et al. (43). The UniRef_90 gene families are regrouped to gene ontology (GO) terms (44) to get an overview of GO functions in the community. The abundance values are normalized using total-sum scaling (TSS) normalization to produce “copies per million” (CPM).

Metagenomic and metatranscriptomic reads from Lake O, CR, and mesocosms were trimmed and filtered for quality and adapter removal using bbduk (45) as described by Krausfeldt et al. (40). Metagenomic reads were assembled in KBase (46) using metaSPAdes v3.15.3 (47) with error correction and kmers 55, 77, and 99. Metagenome-assembled genomes (MAGs) generation, taxonomic and functional annotation, and phylogenetic analysis were performed in KBase. MAGs were recovered from assemblies using the apps in KBase for CONCOCT v1.1 (48), Maxbin2 v2.2.4 (49), and MetaBAT2 v1.7 (50), and then refined with DAS Tool v1.0.2. MAGs were taxonomically annotated using Genome Taxonomy Database Toolkit (GTDB-Tk, 51). The microcystin gene cassette was identified as described by Krausfeldt et al. (40) using a translated search of MAGs with blastx. Phylogenetic analysis was performed with SpeciesTree v2.2.0, which employs FastTree2, with all *Microcystis* MAGs from Lake O and the CR with reference genomes for all *Microcystis* spp. available in KBase. The final tree was pruned in the Interactive Tree of Life (iTol) application (https://itol.embl.de/).

Differential expression analysis was also performed in KBase by aligning reads to reference genomes with HISAT2 v2.1.0, assembling transcript with StringTie v2.1.5, and identifying differentially expressed genes with DESeq2. The reference genomes for *Microcystis panniformis* FACHB-1757 (CP011339.1), *Microcystis* phage MaMV-DC (YP_009217776.1), *Microcystis* virus La-MM01 (NC_008562), and *Microcystis* phage mic1 (MN013189) were used. *M. panniformis* was chosen as the reference genome for *Microcystis* sp. in the mesocosms and CR, because it was identified as the dominant cyanobacteria from both the read annotations from CosmosID-HUB and GTDB-Tk results of *Microcystis* MAGs. EggNOG-mapper v2 (52) was used to annotate the differentially expressed genes from *M. panniformis* with Kyoto Encyclopedia of Genes and Genomes (KEGG). Functional pathway inference was performed by using KEGG Orthology (KO) IDs to assign involvement in pathways from the KEGG database.

## RESULTS AND DISCUSSION

### *Microcystis* ecotype associated with the bloom on the Caloosahatchee River was highly identical to *Microcystis* ecotypes of Lake Okeechobee

**Fig 1:**
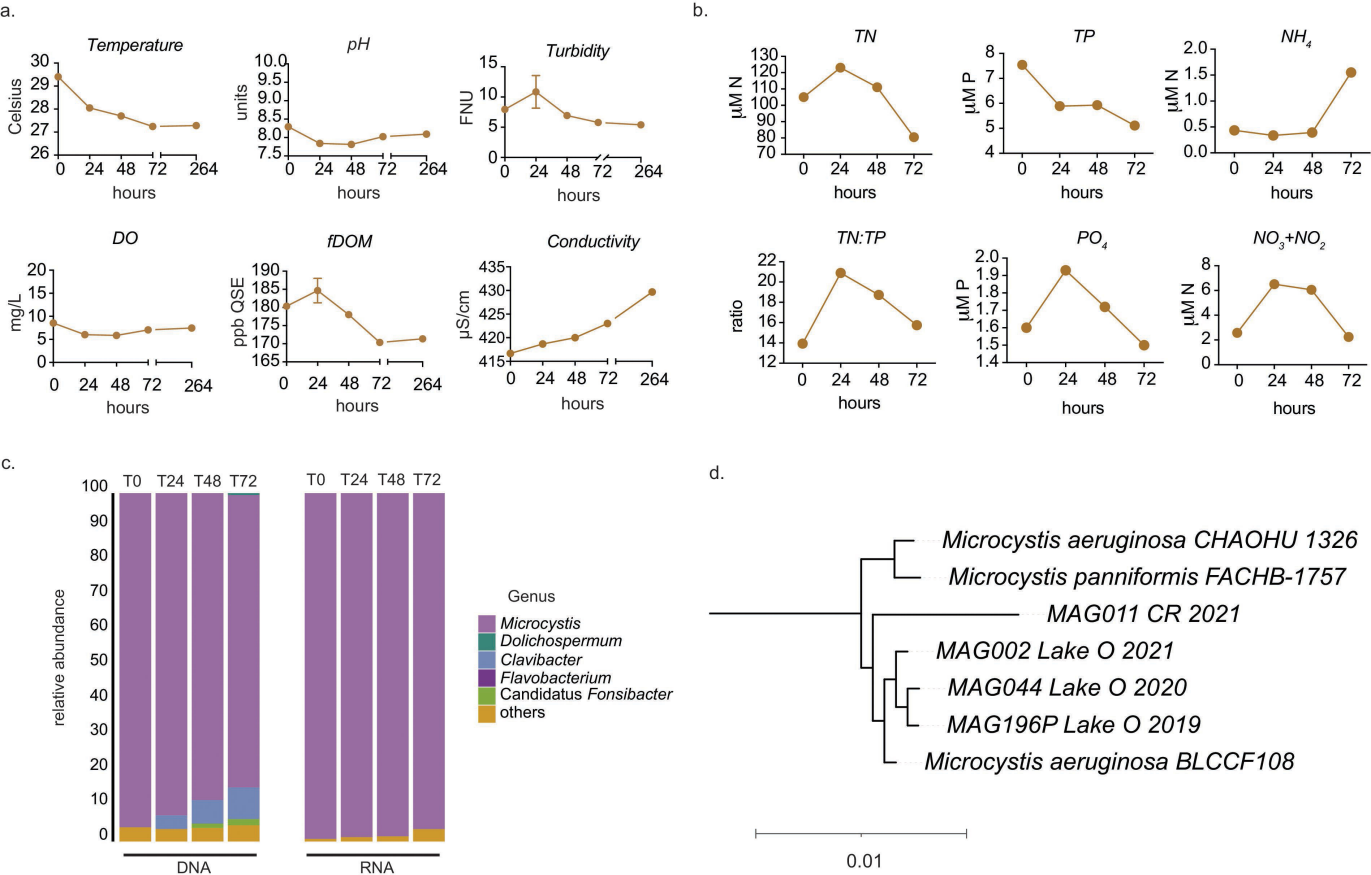
Nutrients, physiochemical parameters, and microbial community composition in CR bloom in May 2021. (**a**) Chemical data collected from the CR during the bloom using YSI. Data represent the average of measurements at the surface, mid, and bottom of the river. Error bars represent standard error. (**b**) Nutrient chemistry in the CR during the bloom. (**c**) Present and active microbial community composition of the CR bloom based on metagenomic (DNA) and metatranscriptome (RNA) reads, respectively. (**d**) Phylogenetic tree showing relationships between Lake O and CR MAGs recovered from 2019 to 2021. MAGs on the tree are labeled where and in what year they were recovered.

At the initiation of the mesocosm experiments on 17 May 2021, samples were collected directly from the *Microcystis* bloom on the CR. The highest concentration of chlorophyll a (chl *a*) measured over the course of the bloom in the CR was 77 µg/L. Suspended algal colonies, predominantly *Microcystis,* peaked at 3.5 × 10^4^ colonies/L. Microcystin-LR was detected at a concentration of 3.8 µg/L. At the initiation of the experiments, water temperature was 29.40°C ± 0.12°C (mean ± standard deviation), conductivity was 416.67 ± 0.58 µS/cm, pH was 8.29 ± 0.12, DO was 8.54 ± 0.55 mg/L, turbidity was 7.94 ± 0.99 formazin nephelometric units (FNU), and fDOM was 484.96 ± 4.27 ppb quinine sulfate equivalents (QSE) (Fig. 1a). Over 72 h, the pH and DO remained relatively constant. In contrast, water temperature, turbidity, and fDOM generally decreased with increased conductivity (Fig. 1a). At the start of the experiments, total nitrogen (TN) and total phosphorus (TP) in the river were at approximately 105 µM N and 7.5 µM P, respectively, equivalent to TN:TP of ~14 (Fig. 1b). PO_4_ concentrations were at ca. 1.5 µM P, and NH_4_ and NO_3_+NO_2_ concentrations at 0.5 µM N and 2.5 µM N, respectively (Fig. 1b).

Annotation of short reads from metagenomic sequencing of samples collected from the CR confirmed that the bloom was dominated by *Microcystis* (Fig. 1c). A MAG was identified as *Microcystis* (MAG011) and contained the entire microcystin biosynthesis gene cassette. MAG011 was >99% identical to three other *Microcystis* MAGs derived from metagenomes from Lake O. Together, this formed a clade with *Microcystis aeruginosa* BLCCF108, which was also isolated in Lake O in 2019 (53). Additional clade members included *M. aeruginosa* CHAOHU 1326 from Chao Lake in China and *M. panniformis* FACHB-1757 from Lake Taihu in China (Fig. 1d; Fig. S1) (54, 55). Based on GTDB, the MAGs from this study that were in this clade were most closely related to *M. panniformis*_A (*M. panniformis* FACHB-1757 according to NCBI’s taxonomy). This falls within a clade with other *M. aeruginosa* genomes, suggesting this species classification may be incorrect. Two other *Microcystis* MAGs identified (MAG163 and MAG196) were more distantly related to this group. This indicates that different lineages of *Microcystis* exist in Lake O (Fig. S2). However, the lineage including MAG011 persisted across multiple years in Lake O, leading up to the bloom on the CR. This included two previous years (2020 and 2021) when blooms were significant on Lake O.

These observations suggest the *Microcystis* lineage forming CR blooms was the same as that causing the Lake O blooms. Anthropogenic influences on the CR are substantial, with watershed nutrient loads equal to or greater than Lake O’s (56–59). The influence of Lake O outflows on the ability of the lineage to bloom in the CR is not clear from this study. Considering the difference in physical and chemical properties between Lake O and CR, the factors causing *Microcystis* blooms of this lineage in either location may be distinct. Furthermore, these MAGs may represent different ecotypes that are endemic to the area. Future work to close these genomes and monitor the presence and persistence of *Microcystis* lineages and/or ecotypes will be valuable to elucidate the role of outflows from Lake O on the presence of *Microcystis* blooms in the CR.

### N additions maintained and increased chlorophyll *a*, phycocyanin, and bloom duration

Mesocosm experiments were initiated from water with visible surface scum from the CR. PO_4_ or urea (as a source of N) was added to the *in situ* mesocosm chambers at time T0, T24, and T48. We chose to use urea as a source of N as its usage in agriculture has increased nearly 100-fold over the past four decades (60), and it has been detected in lakes at high concentrations as agricultural runoff (16). Owing to its chemical structure (CH_4_N_2_O), it serves as both an N and C source for *Microcystis* (61). In Florida, urea is particularly important as it is an agricultural fertilizer for citrus crops, among other agricultural practices, and has been detected in Lake O and the CR (62–64). However, few studies have investigated the impact of urea on cyanoHABs in the CR, although the total dissolved pool of N has been shown to be largely made up of organic sources of N (64). As a control, mesocosms without the addition of nutrients were included. As expected, increases in PO_4_ and urea were observed 1 hour after their addition (Fig. S3). After the final addition of nutrients, 300 µM N was added to the urea treatment and 30 µM P to the PO_4_ treatment.

**Fig 2:**
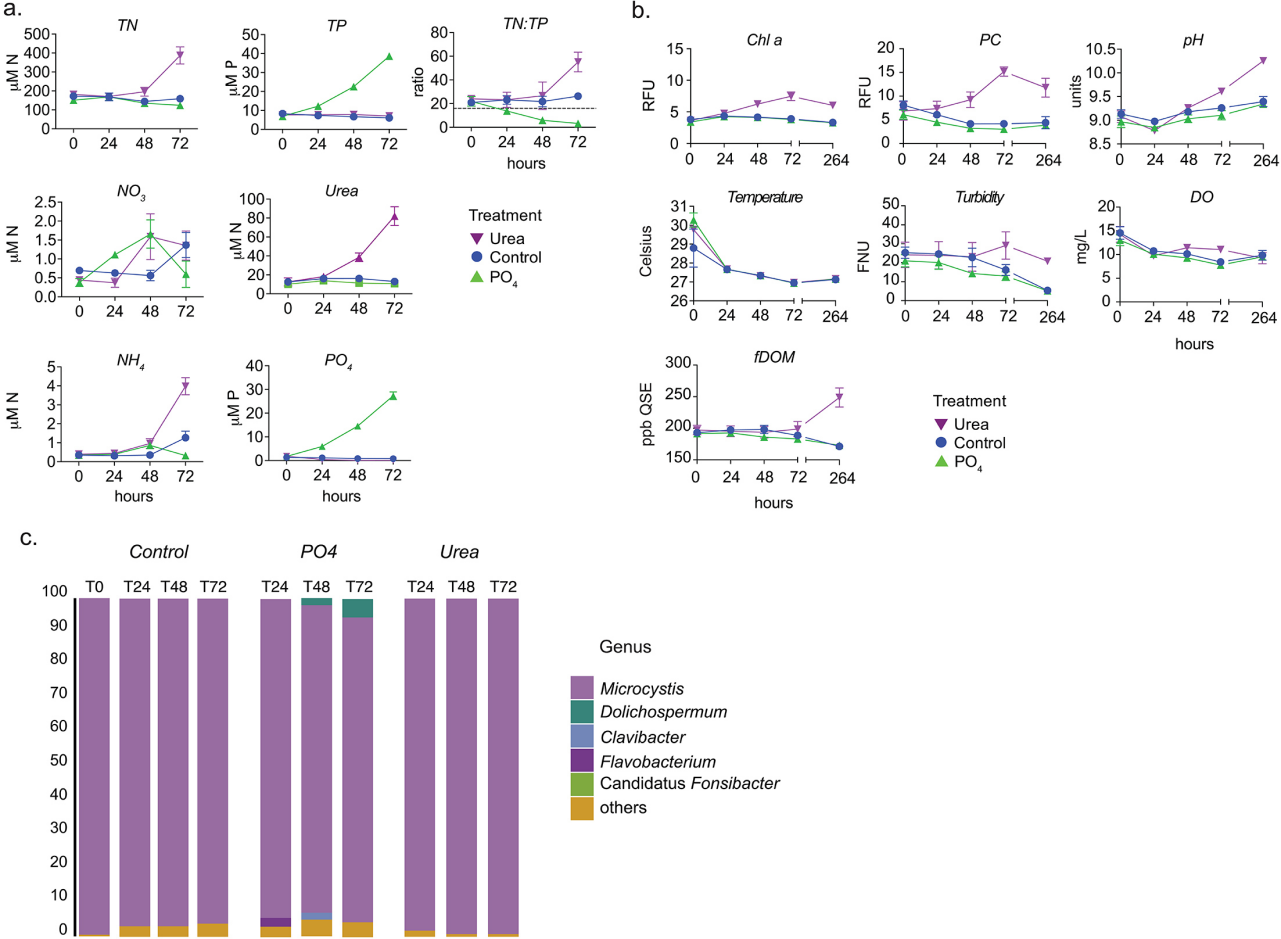
Nutrients, physiochemical parameters, and active communities in mesocosms of the Caloosahatchee River. (**a**) Nutrient concentrations in mesocosm chambers at the surface at the start of experiments (T0), after 24 hours (T24), after 48 hours (T48), and after 72 hours (T72). Nutrients were added at increasing concentrations at T0, T24, and T48, and samples for nutrient concentrations were collected before nutrient addition. Samples for dissolved nutrient analysis were also collected after nutrient addition (Fig. S3). For panels a and b, data show the average measurement of three biological replicates and error bars represent standard error. (**b**) Chemical profiles for mesocosm chambers at the surface at T0, T24, T48, T72, and 1 week later at T264. Chemical profiles were prepared before nutrient dosing that occurred at T0, T24, and T48. (**c**) The top 95% of the taxonomic composition of the metatranscriptomes (active microbial community).

The nutrient addition to the mesocosm successfully altered nutrient dynamics in the chambers. The addition of urea increased TN compared to all other treatments (two-way analysis of variance [ANOVA], *P* < 0.0001). Based on the Redfield ratio, this rendered the environment P-limited by T72 (Fig. 2a) (65). The PO_4_ treatment was the opposite; TP increased compared to the other treatments and control (two-way ANOVA, *P* < 0.0001). The addition of PO_4_ made the environment N limited (Fig. 2a). Urea was consumed, with 82 µM urea accumulating in the urea treatment. However, PO_4_ accumulation indicated little consumption. This suggested the CR bloom was N limited and P replete, although TN:TP in the river suggested a balance in N or P requirements.

The addition of N in the form of urea had the greatest impact on chl *a* and phycocyanin (PC) by T72 (Fig. 2b, two-way ANOVA, *P* < 0.0001). Urea additions had a lasting effect on the bloom, maintaining higher chl *a* and PC compared to the control (two-way ANOVA, *P* < 0.0001) (Fig. 2b). Interestingly, none of the nutrient additions significantly affected turbidity compared to the control over 72 hours. However, turbidity was significantly greater for mesocosms treated with urea than all other nutrients at T264 (two-way ANOVA, *P* < 0.035). A similar trend was observed for fDOM, where urea-treated mesocosm had significantly greater values than the control and PO_4_ additions at T264 (two-way ANOVA, *P* < 0.0001). This suggests that chl *a* and PC synthesis were enhanced over 72 hours in the mesocosm treated with urea. The significantly greater fDOM and turbidity in urea-treated mesocosms at T264 is consistent with the aforementioned lasting effect of urea addition on bloom persistence. No impact on the bloom was noted by proxy of chl *a* and PC in PO_4_ treatment. Accordingly, the addition of N in the form of urea, but not PO_4_, potentiated the persistence of the bloom.

Gene expression indicated that nutrient additions also altered the microbial community. Gene expression from *Microcystis* was dominant in the control and in both urea- and PO_4_-treated mesocosms (Fig. 2c). In the control mesocosm (no nutrient addition), minor increases in expression from “other” taxa were observed over time. In contrast, there were fewer gene expression changes in the urea-treated mesocosm, as “other” taxa were comparable to T0 in the control. This may indicate that adding N allows *Microcystis* to outcompete other community members by suppressing their abundance or limiting their ability to perform transcription. In the PO_4_-treated mesocosms, changes in gene expression were noted throughout the experiment. At T24, expression from members of the *Flavobacterium* genus increased. This was short-lived, however, as expression was reduced at T48 and T72. Interestingly, the reduction in expression from *Flavobacterium* corresponded with an increase in the N-fixing cyanobacterium *Dolichospermum* [linear discriminant analysis (LDA) effect sizes (LefSe), LDA score = 4.440, *P* = 0.016 at T72]. We also noted an increase in “other” taxa at T24, T48, and T72 relative to T0 in the control. Thus, adding PO_4_ permitted gene expression from other community members at the expense of dominance by *Microcystis*. When considering the impact that PO_4_ and urea had on bloom duration (Fig. 2b), it is possible that gene expression from taxa other than *Microcystis* limited bloom formation in the PO_4_-treated mesocosm. Otherwise, when *Microcystis* dominated in the urea-treated mesocosm, the lack of gene expression from competing taxa may have potentiated the duration of the bloom. This suggests that competing microbial taxa may limit bloom duration by *Microcystis* even in the presence of anthropogenically introduced nutrients.

### Transcriptional response to nutrients by *Microcystis*

**Fig 3:**
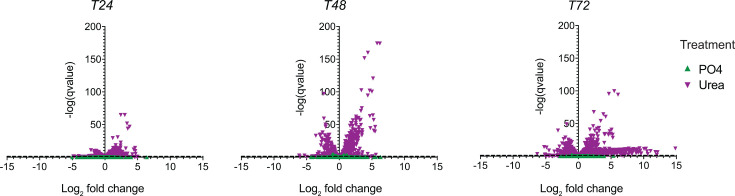
Differential expression of *Microcystis* in nutrient-amended mesocosm chambers. Volcano plots depicting significantly up- and down-regulated genes of *Microcystis* according to nutrient treatment compared to the control at each time point. q-value represents the *P* value after corrections for multiple comparisons using a false discovery rate. Log_2_ fold change represents the effect size and direction. The -log(qvalue) represents statistical significance, thus the higher on the y-axis the more significant the effect.

Given the dominance of *Microcystis* in all three mesocosms, we sought to understand how gene expression changes with and without the addition of nutrients. PC and chl *a* changes corresponded to the global transcriptional response of *Microcystis*. At T24, T48, and T72 for urea additions, *Microcystis* demonstrated 75, 161, and 916 differentially expressed genes, respectively, relative to the control (Fig. 3; Data S1). A transcriptional response to PO_4_ was not observed, suggesting that PO_4_ did not directly affect *Microcystis*.

**Fig 4:**
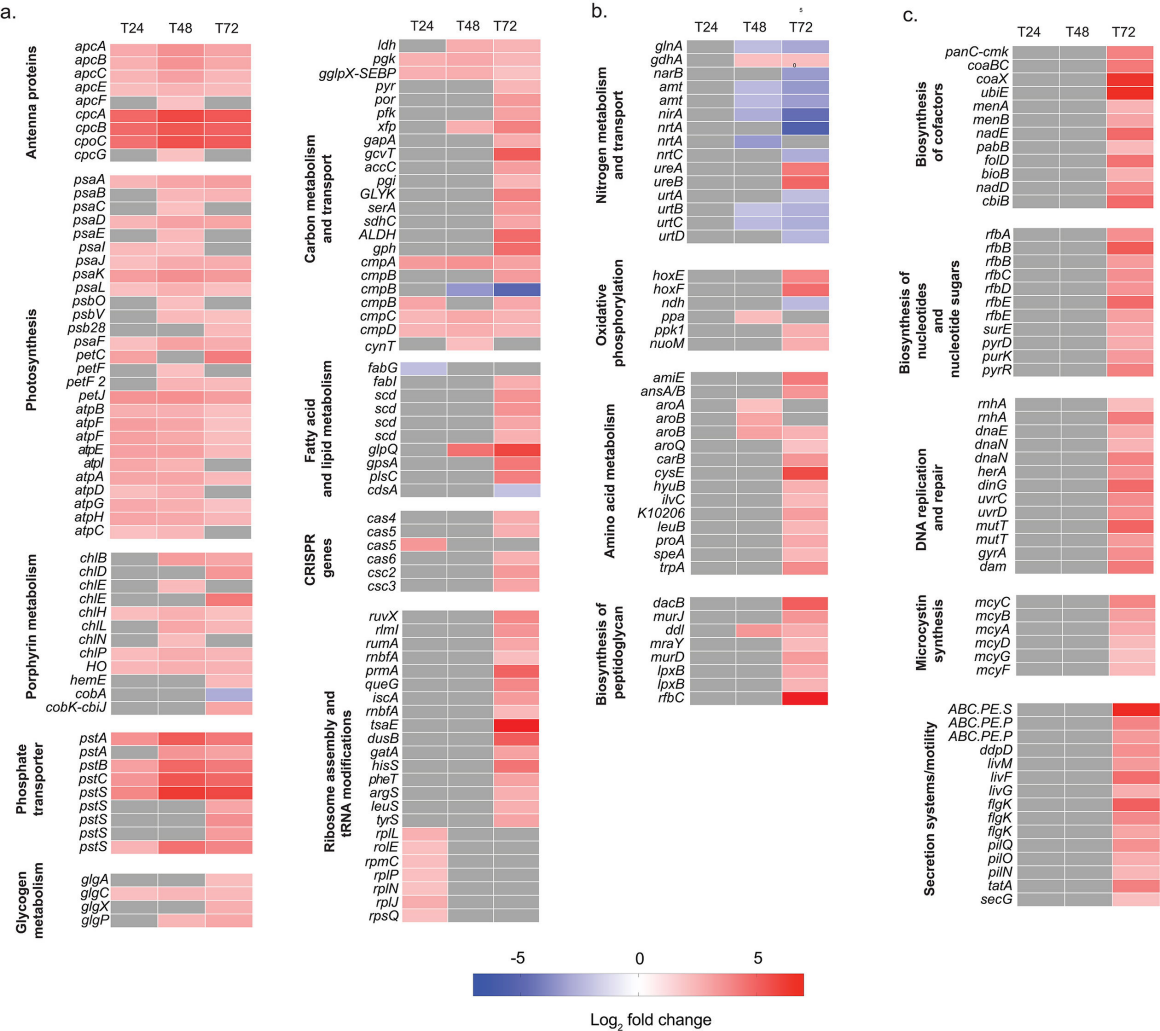
Differentially expressed genes that were statistically significant in the urea-amended treatment at 24 hours (T24), 48 hours (T48), and 72 hours (T72). Cutoffs for statistical significance were q values (p values after correction for multiple comparisons) of <0.05 and log_2_ fold change greater than 2 or less than −2. Log_2_ fold change represents the effect size and direction. Genes are gray at timepoints where they did not meet the criteria. Duplicated genes are copies of that gene.

Based on differential gene expression, the addition of urea resulted in the maintenance of photosynthesis, pigment production, PO_4_ and CO_2_ scavenging, and ribosome restoration. Urea addition (50 µM N) after 24 hours corresponded to the upregulation of phycocyanin (*cpcABC*), allophycocyanin (*apcABCE*), porphyrins (*chlHP, HO*), photosystem I (*psaADFIJKL*), ATP synthase (*atpABCDEFGH*), electron transport (*petJ*), cytochrome 6b/f (*petC*), PO_4_ transport genes (*pstABCS*), inorganic C transport (*cmpABCD*), and glycolysis (*pgk, glpX*) coding genes, and those encoding ribosomal biosynthesis, namely, *rplEJLNP, rpmC,* and *rpsQ* (Fig. 4). Glycogen synthesis (*glgC*) was also upregulated (Fig. 4).

Previous studies showed that N limitation is associated with the downregulation of photosynthesis machinery and C and amino acid metabolism (66, 67). Our results indicate that these genes are upregulated with the addition of N, and those functions are first to be restored. Although PO_4_ transporters in *Microcystis* are expressed during P limitation (68), the results of this study indicate regulation by N availability. Thus, without sufficient N, PO_4_ uptake is likely reduced.

These biological functions continued with each urea addition. At T48, 24 hours after the second round of urea (100 µM N), the additional consumption of ~50 µM of N corresponded with upregulation of genes encoding photosystem II (*psbOV*), antenna proteins (*cpcAG*), photosystem I (*psaBCE*), electron transport (*petF*), and porphyrin metabolism (*chlBELN*), in addition to genes already upregulated at T24 (Fig. 4). All PO_4_ transporters remained upregulated at T48, as did genes involved in central C metabolism (*ldh, xfp*) and sulfate permease (*sulP*) (Fig. 4).

An effect on N metabolism was noted at T48. Notably, NO_3_, NH_4_, and urea transporters (*nrtAC, amt,* and *urtABCD*, respectively) were downregulated. NO_3_ metabolism genes (*nirA, narB*) and glutamine synthetase (*glnA*), a key enzyme in NH_4_ assimilation, were downregulated at this time (Fig. 4). This coincided with previous studies that showed N transporters in *Microcystis* are upregulated during N limitation and downregulated when N requirements are met (67, 69). Glutamate dehydrogenase was upregulated, which converts NH_4_ directly into glutamate, supporting a shift in N flux through metabolism.

At T72, 24 hours after a third addition of urea (150 µM N), an increased accumulation of urea was observed. Genes for NH_4_ transport, urea transport, and glutamine synthetase remained downregulated. Glutamate dehydrogenase was still upregulated. Additional NO_3_ transport (*nrtAC*) and metabolism (*narB*) genes were downregulated. Genes involved in urea transport (*urtAD*) were also downregulated. Genes encoding urease (*ureAB*, Fig. 4), which are responsible for the catabolism of urea, were upregulated. Genes encoding photosystem II (*psb28*), porphyrins (*hemeE, cobK, chlDE*), PO_4_ transporters (three additional copies of *ptsS*), central C cycling genes (*pyr, por, pfk, gapA, gcvT, accC, pgi, GLYK, serA, sdhC, ALDH, gph*), and inorganic C transporters (*cmpB*) continued to be upregulated. This indicates that *Microcystis* was likely still scavenging P and C and using urea (Fig. 4).

Based on increased PC and chl *a* at T48 and T72, new pathways upregulated likely played an important role in supporting bloom photosynthesis once cells recovered from N limitation. At T72, *Microcystis* metabolism shifted to using N for other cell processes. These included genes involved in cell replication and growth, including amino acid metabolism (*amiE, ansAB,* additional copies of *aroB, aroQ, carB, cysE, hyuB, ilvC, leuB, proA, speA, trpA*), peptidoglycan biosynthesis (*dacB, murJ, mraY, murD, lpxB, rfbC*), fatty acid biosynthesis (s*cd, fabI*), glycerolipid metabolism (*gpsA, plsC*), biosynthesis of vitamins (*pamC-cmk, coaBCX, ubiE, menAB, pabB, bioB, cbiB*) and other cofactors (*nadDE, folD*), metabolism of nucleotides and their sugars (r*fbACDE, surE, pyrD, purK, pyrR*), and DNA replication and repair (*rnhA, dnaEN, herA, dinG, uvrCD, mutT, gyrA, dam*). Interestingly, oxidative phosphorylation genes, including those involved explicitly in electron flow (*hoxEF, nuoM, ndh),* were also differentially expressed. This suggested that the redox status of the cells changed, indicating transcriptional changes translated to cell activity.

Taken together, the addition of N in the form of urea initially resulted in the upregulation of genes involved in photosynthesis and C, P, and N acquisition. This suggests that *Microcystis* was utilizing the addition of nutrients to increase activity in these metabolic pathways. The upregulation of genes involved in photosynthesis is consistent with the increase in chl *a* and PC that we observed in mesocosms. However, once the N requirement was fulfilled, as evidenced by a reduction in expression of genes associated with N uptake, gene expression shifted to other metabolic pathways, such as those involved in amino acid, lipid, vitamin, cofactor, and DNA synthesis. As N is critical for microcystin production (70), *Microcystis* can also expend energy on microcystin once N requirements are fulfilled.

### N additions corresponded to genomic rearrangement and phage defense

**Fig 5:**
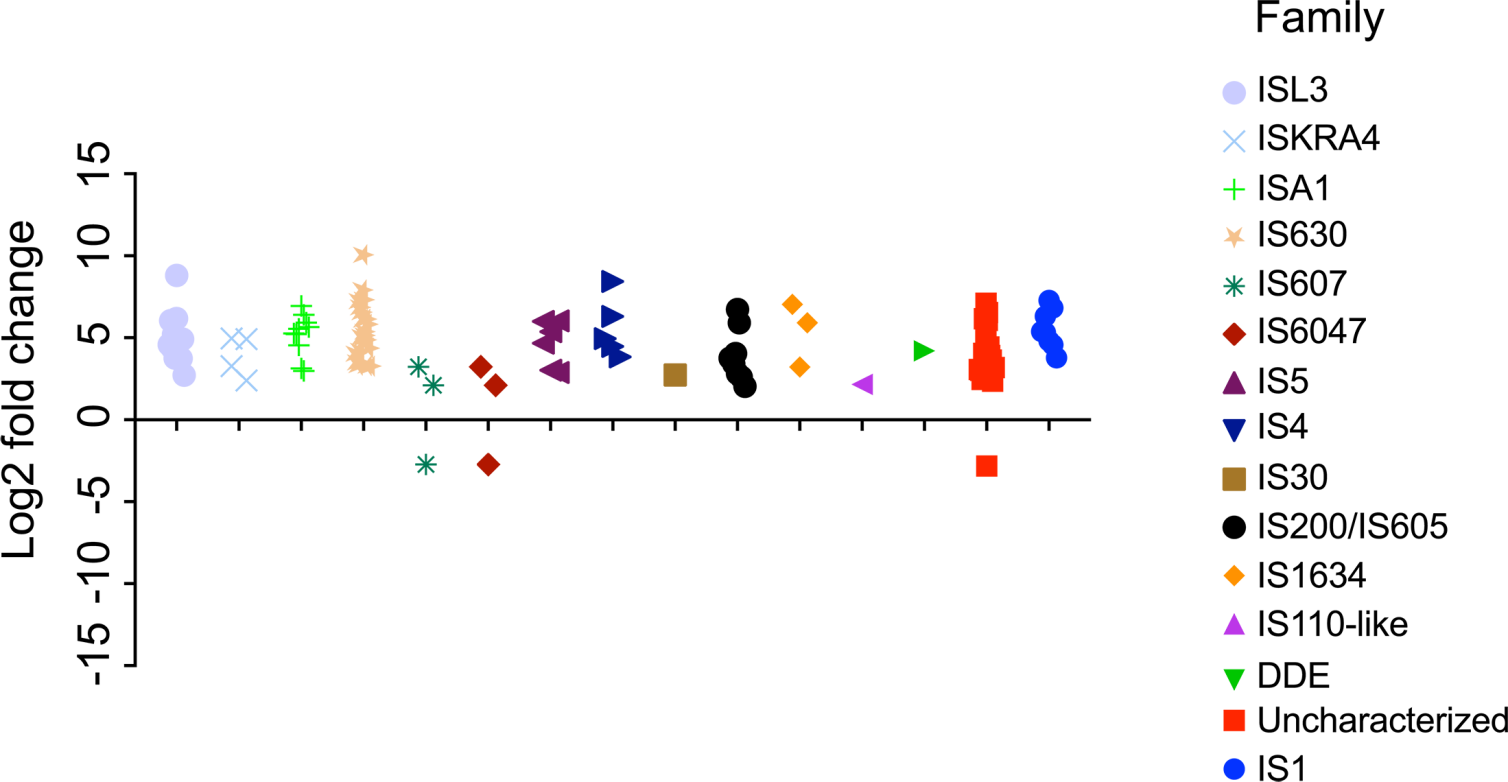
Differentially expressed transposase families in the urea-amended treatment at 72 hours (T72). Each symbol represents a differentially expressed putative transposase, with colors corresponding to its predicted transposase family.

The upregulation of many transposases by *Microcystis* at T72 was perhaps the most striking (Fig. 5; Data S2). Over 100 differentially expressed *Microcystis* genes were annotated as transposases of several different families, including IS1, IS100, IS1634, IS200/IS605, IS30, IS4, IS5, IS607, IS630, ISAs1, ISKra4, ISL3, and ISNCY (Fig. 5). Most were not well characterized. Only 20 had associated KEGG orthologs confirming their function as transposases. *Microcystis* and other bloom-forming cyanobacteria are known to have transposase-enriched genomes (71, 72), with a hypothesized role in prevailing environmental conditions (69, 73, 74), including nutrient availability. Transposition, mediated by transposases, plays an important role in horizontal gene transfer, genome rearrangement, and adaptation during stressful conditions (75, 76). Using laboratory cultures, Steffen et al. (74) showed that, under N-limiting conditions and using urea as the N source, many transposases are upregulated in *M. aeruginosa*. In addition, Harke et al. (69) showed that under *in situ* conditions, transposases were upregulated by N, including urea, but more intensely by the addition of PO_4_. Thus, transposase activity in *Microcystis* has been attributed to N limitation. The results here suggest most transposases are upregulated when conditions are N replete. Transposase activity has also been linked with the attachment of bacteria, such as those in biofilms or particles, as a mechanism for genetic exchange within the community (77, 78). Indeed, *Microcystis* upregulated genes encoding functions to promote colony formation, such as motility (*flgK*), secretion (*pilNOQ, tatA, secG*), quorum sensing (ABC.PE.S, ABC.PE.P, *ddpD, livFGM*), and transposition (Fig. 4). Together, this suggests there are specific densities or cues for *Microcystis* to undergo genomic rearrangement to promote heterogeneity within the community.

Upregulation of genes encoding several families of *Microcystis* Type II toxin/antitoxin (TA) systems was observed with urea addition at T72 (Table 1). Most were uncharacterized, with seven having associated KEGG orthologs. However, an additional 10 were identified that were previously annotated on the genome. These results suggest most genes encoding toxin and antitoxin production were upregulated, with only a few downregulated. Although it is known that cyanobacteria possess many TA systems, their physiological function in *Microcystis* is unclear. Type II TA systems encode two proteins, a toxin and an antitoxin, which are prevalent on mobilizable genetic elements and found on the chromosomes of many diverse bacterial taxa (79). When they occur on mobilizable genetic elements, such as plasmids, they help stabilize plasmid maintenance (80). Their role, however, when integrated into chromosomes is less clear, with some suggesting that they contribute to the formation of persister cells (81), stress response (82), and stabilization of areas of the genome (83). Interestingly, recent evidence suggests that TA systems may function to prevent phage infection (84). For example, the deletion of the MazF/MazE type II TA system resulted in increased phage production (85). TA systems have been shown to result in host metabolic dormancy upon entry of foreign DNA (84) and are thought to protect neighboring cells from infection.

**TABLE 1:** Differentially expressed genes in urea treatment identified as Type II toxin-antitoxin systems based on KO IDs or *Microcystis* genome annotations.

Gene_ID	Gene description	KEGG orthology	KEGG gene	T24 log2 fold change	T48 log2 fold change	T72 log2 fold change
VL20_RS24925	Type II toxin-antitoxin system HicA family toxin					6.35
VL20_RS30550	Type II toxin-antitoxin system HicA family toxin					5.81
VL20_RS30215	Type II toxin-antitoxin system HicA family toxin					5.92
VL20_RS30545	Type II toxin-antitoxin system HicA family toxin					4.60
VL20_RS24930	Type II toxin-antitoxin system HicB family antitoxin					5.63
VL20_RS24990	Type II toxin-antitoxin system HicB family antitoxin					3.85
VL20_RS25250	Type II toxin-antitoxin system HicB family antitoxin					6.22
VL20_RS23405	Type II toxin-antitoxin system HicB family antitoxin					3.33
VL20_RS30515	Type II toxin-antitoxin system HicB family antitoxin					6.83
VL20_RS30140	Type II toxin-antitoxin system HicB family antitoxin					2.58
VL20_RS10820	Type II toxin-antitoxin system HicB family antitoxin					−2.33
VL20_RS17915	HicB family protein					−4.56
VL20_RS30680	Type II toxin-antitoxin system mRNA interferase toxin	K06218	*relE*			6.69
VL20_RS23930	Type II toxin-antitoxin system PemK/MazF family toxin	K07171	*mazF*			5.29
VL20_RS00520	Type II toxin-antitoxin system Phd/YefM family antitoxin	K19159	*yefM*	−2.38		−2.08
VL20_RS22020	Type II toxin-antitoxin system Phd/YefM family antitoxin					2.92
VL20_RS25270	Type II toxin-antitoxin system RelE/ParE family toxin	K19092	*paeE*			4.56
VL20_RS23075	Type II toxin-antitoxin system RelE/ParE family toxin	K07334	*higb-1*			2.77
VL20_RS25590	Type II toxin-antitoxin system RelE/ParE family toxin					2.88
VL20_RS24555	Type II toxin-antitoxin system VapC family toxin	K18828	*vapC*			5.07
VL20_RS22015	Type II toxin-antitoxin system VapC family toxin	K07062	*fitB*			3.50
VL20_RS25580	Type II toxin-antitoxin system VapC family toxin	K07065				3.60
VL20_RS21115	Type II toxin-antitoxin system VapC family toxin					4.28
VL20_RS21110	Type II toxin-antitoxin system VapC family toxin					4.30
VL20_RS29815	Type II toxin-antitoxin system VapC family toxin					3.61
VL20_RS31490	Type II toxin-antitoxin system VapC family toxin				−2.44	
VL20_RS26225	PIN domain-containing protein	K18828	*vapC*			6.96
VL20_RS20925	PIN domain-containing protein	K18828	*vapC*			2.06
VL20_RS30500	Hypothetical protein	K18828	*vapC*			4.49
VL20_RS29845	VapC toxin family PIN domain ribonuclease		*VapC*	2.51		2.51
VL20_RS23250	Type II toxin-antitoxin system MqsA family antitoxin					8.17
VL20_RS24910	Antitoxin family protein					3.72
VL20_RS23080	HigA family addiction module antidote protein	K21498	*higA*			4.25
VL20_RS23085	Hypothetical protein	K19155	*yhaV*			3.74
VL20_RS23915	Hypothetical protein	K06218	*relE*			5.89
VL20_RS22740	Hypothetical protein	K07746	*parD1/3/4*			4.68
VL20_RS25265	Hypothetical protein	K07746	*parD1/3/4*			4.41
VL20_RS00515	Txe/YoeB family addiction module toxin	K19158	*yoeB*			−2.82
VL20_RS22995	Type II toxin-antitoxin system death-on-curing family toxin	K07341	*doc*			4.20
VL20_RS02055	Type II toxin-antitoxin system mRNA interferase toxin				−2.80	

Previous work has shown that activation of CRISPR, a well-known phage defense system, is induced in *Microcystis* upon phage infection (86, 87). Interestingly, several CRISPR genes became upregulated with the addition of urea, including *cas5* at T24 and *cas4, cas5, cas6, cas7,* and *cas10d* at T72. Along with the upregulation of TA systems, this suggested *Microcystis* responded to phage infection. Gene expression analyses using publicly available reference phage genomes showed ongoing phage infection in all sampled chambers (Fig. S4) but no difference in phage expression for any of the treatments. However, specific upregulated CRISPR genes encode key proteins for integrating functional spacers and forming effector complexes to degrade foreign DNA (88, 89). Thus, *Microcystis* incorporated spacers as immunological memory and to actively fight ongoing phage infection. Concordant upregulation of endonucleases and methylases at T72 (Data 1), which are also involved in host defense, supports this notion (90, 91).

Overall, the collective upregulation of TA and phage defense systems at T72 suggested that *Microcystis* increased the ability to protect itself against phage, contributing to the persistence of the bloom observed at T264. The notion that increased expression of transposases may facilitate genomic rearrangement and increase genetic heterogeneity may also help to ensure a sustained bloom. Indeed, previous work has found that increased mutation rates and the resulting genetic heterogeneity can help bacterial populations survive unpredictable and stressful environments (92).

### Expression of microcystin biosynthesis genes induced by urea

Based on the increased expression of genes involved in synthesizing microcystin, N is likely to stimulate microcystin biosynthesis. Upregulation of *mcyABCDGF* genes in the *mcy* operon encoding microcystin biosynthesis, including peptide synthesis and modification, was observed at T72 (Fig. 4). Although microcystin concentrations were unavailable for this study, these results agree with those of other studies. For example, high N loads in freshwater ecosystems have been hypothesized to help drive the production of microcystins (93). Urea as an N source has been shown to increase microcystin biosynthesis compared to other N sources (94), such as NO_3_ and NH_4_. This may be because urea can also be used as a source of C (61). However, the underlying mechanism of microcystin production has remained elusive. This is owing to the difficulty in genetically modifying *Microcystis*, and the many conditions associated with microcystin production (95).

Many other factors have been attributed to the upregulation of *mcy* genes. The urea additions strongly increased the TN:TP, which has also been shown to increase microcystin production (96). Greater amounts of microcystin have also been observed with higher TC:TN or C and N imbalance (97). Some studies have proposed that microcystin plays a role in combating oxidative stress (98, 99). Interestingly, the gene expression profiles presented herein indicated a urea-enhanced photosynthetic activity. This could generate oxidative stress and trigger the upregulation of genes encoding microcystin biosynthesis. Concordantly at T72, gene expression also suggested a shift in the redox state, indicating the cells were experiencing oxidative stress. Recent modeling of microcystin biosynthesis in *Microcystis* demonstrated the mechanisms triggering microcystin biosynthesis to be more complicated (95). However, the results of this study supported some of the underlying mechanisms that regulate microcystin production. Hellweger et al. (95) showed glyceraldehyde 3-phosphate (G3P) to be one of the limiting metabolites in microcystin biosynthesis. Indeed, with additions of urea in the field study reported here, there was a significant upregulation of several genes encoding glycolysis, including *gapA* (encoding for G3P dehydrogenase), which catalyzes the reversible phosphorylation of G3P. Another metabolite that was identified to be limiting in the report by Hellweger et al. was glutamate. In this study, the upregulation of glutamate dehydrogenase, which produces glutamate, was observed. Thus, increased N availability in the form of urea likely increases microcystin in the CR when a certain threshold is met to shift N demands. However, this phenotype may not be specifically related to growing on urea. Alternatively, it may be linked to specific cellular metabolic flux induced by different environmental cues, like N availability.

In conclusion, we found that urea, but not PO_4_, led to the persistence of a bloom. Upon initial introduction of urea, *Microcystis* increased the expression of genes involved in photosynthesis and N, P, and C capture. However, once *Microcystis* became N replete, downregulation genes involved in N assimilation were observed. This coincided with an increase in the expression of genes involved in generating essential metabolites and DNA, phage defense, and microcystin production, all of which could provide protection and a competitive benefit to *Microcystis*. Together, these changes in transcription may support bloom persistence and eventual intensification, and ensure its longevity beyond the introduction of N associated with anthropogenically introduced chemicals.

## Data Availability

All metagenome and metatranscriptome data are available under BioProject PRJNA813570. YSI data are publicly available at https://doi.org/10.5066/P9JX9NA1.

## SUPPLEMENTAL MATERIAL

Data S1 spectrum.01369-24-s0001.xlsx Differential gene expression in urea treatment depicted in Figure 4.

Data S2 spectrum.01369-24-s0002.xlsx Differentially expressed transposases in urea treatment at all time points corresponding to results shown in Figure 5.

Supplemental material spectrum.01369-24-s0003.pdf Fig. S1 to S4; Tables S1 to S3.

ASM does not own the copyrights to Supplemental Material that may be linked to, or accessed through, an article. The authors have granted ASM a non-exclusive, world-wide license to publish the Supplemental Material files. Please contact the corresponding author directly for reuse.
