# Supplementary material for: Transcriptional profiles of Microcystis reveal gene expression shifts that promote bloom persistence in in situ mesocosms
Source: Microbiol Spectr. 2024 Nov 18;13(1):e01369-24. doi: 10.1128/spectrum.01369-24 (PMC11705957; doi:10.1128/spectrum.01369-24)
Supplement: Supplemental material — Fig. S1 to S4; Tables S1 to S3. [file spectrum.01369-24-s0003.pdf]

Supplemental Figures and Tables

Transcriptional profiles of *Microcystis* reveal gene expression shifts that promote bloom persistence in *in situ* mesocosms

Lauren Krausfeldt[1*], Paisley Samuel[1], Robert P. Smith[1,2,3], Hidetoshi Urakawa[4], Barry H. Rosen[4], Rita Colwell[5], and Jose V. Lopez[1]

1. Department of Biological Sciences, Guy Harvey Oceanographic Center, Nova Southeastern University, Dania Beach, FL
2. Cell Therapy Institute, Kiran Patel College of Allopathic Medicine, Nova Southeastern University, Fort Lauderdale, FL
3. Department of Medical Education, Kiran Patel College of Allopathic Medicine, Nova Southeastern University, Fort Lauderdale, FL
4. Department of Ecology and Environmental Studies, Florida Gulf Coast University, Fort Myers, FL
5. Institute for Advanced Computer Studies, University of Maryland College Park, College Park, MD

*Corresponding author

Dr. Lauren E. Krausfeldt, PhD
Guy Harvey Oceanographic Center
Nova Southeastern University
8000 N. Ocean Drive
Dania Beach, FL 33004
lkrausfe@nova.edu
lkrausfe@gmail.com

a b c

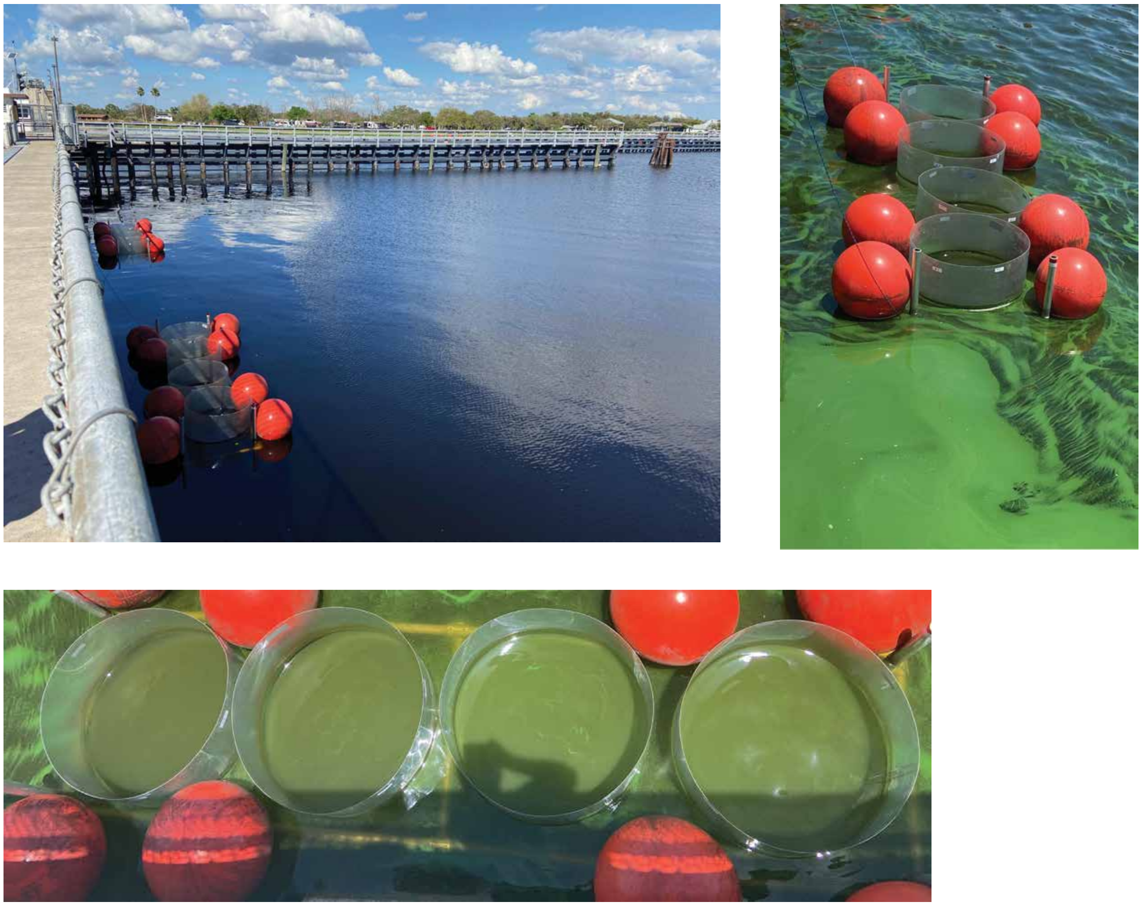

Figure S1. The mesocosm on the Caloosahatchee River at the S-79 lock. (a) The mesocosm chamber set up along the lock. Photo taken in February 2021. The chambers were disassembled, cleaned, and set up in the same orientation for experiments in May 2021. (b) A closer side-view at a group of four mesocosm chambers and conditions at the start of the experiment in May 2021. (c) Above-view of the mesocosms and conditions at the start of the experiments in May 2021.

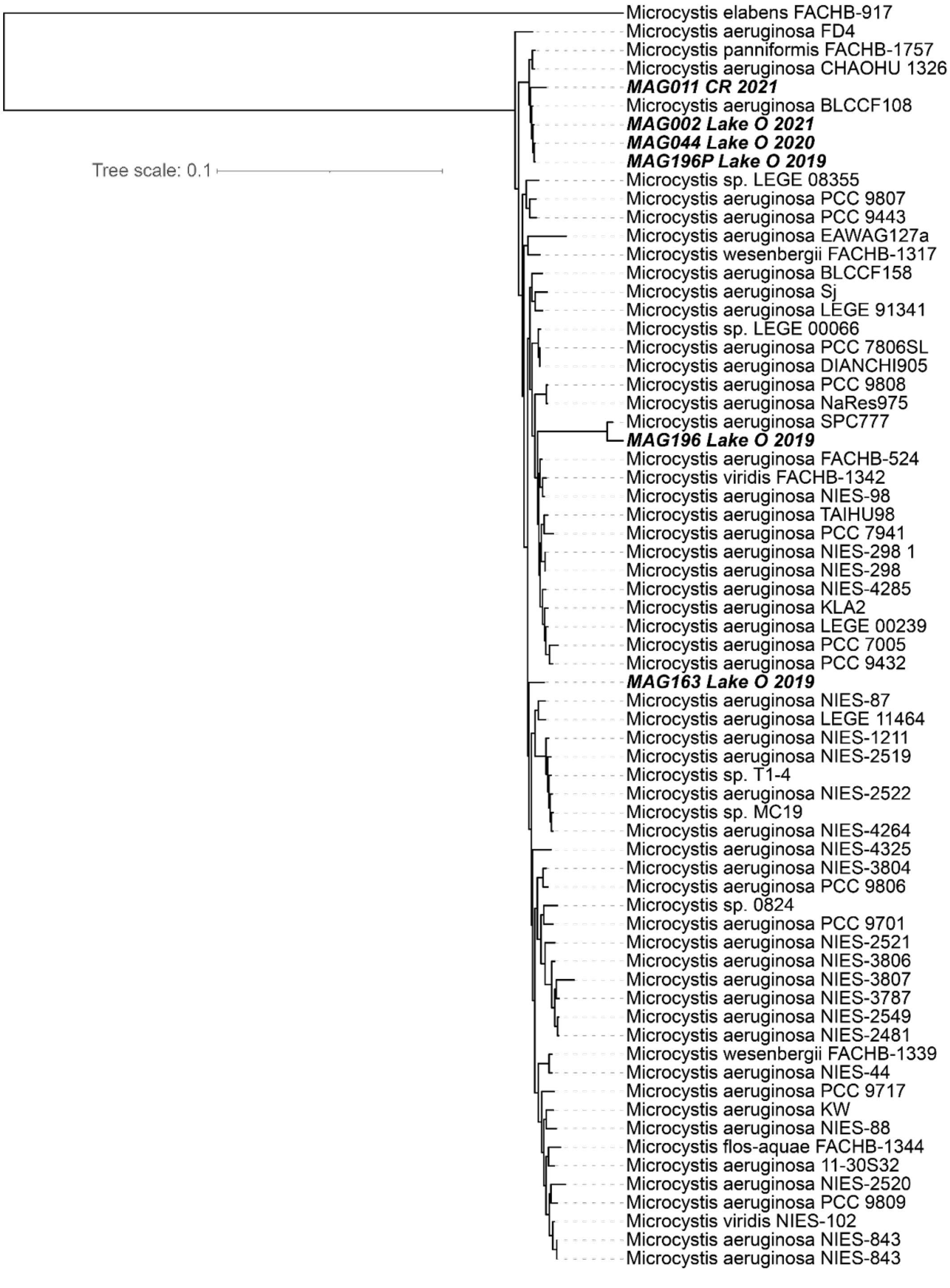

Figure S2. Maximum likelihood phylogenetic tree built from publicly available reference genomes available on KBase of *Microcystis* and metagenome assembled genomes (MAGs) recovered from Lake Okeechobee in 2019, 2020, and 2021 and the Caloosahatchee River during the bloom in May of 2021.

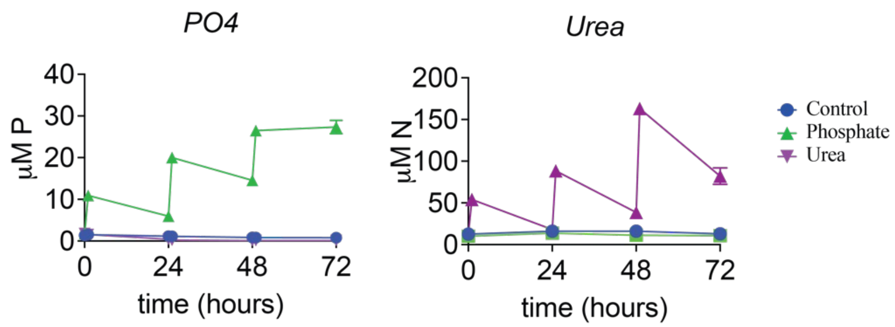

Figure S3. Nutrient concentrations in the mesocosm chambers with measurements taken at ~ 0.5 h, 25 h, and 49 h to capture the addition of PO_4 and urea daily.

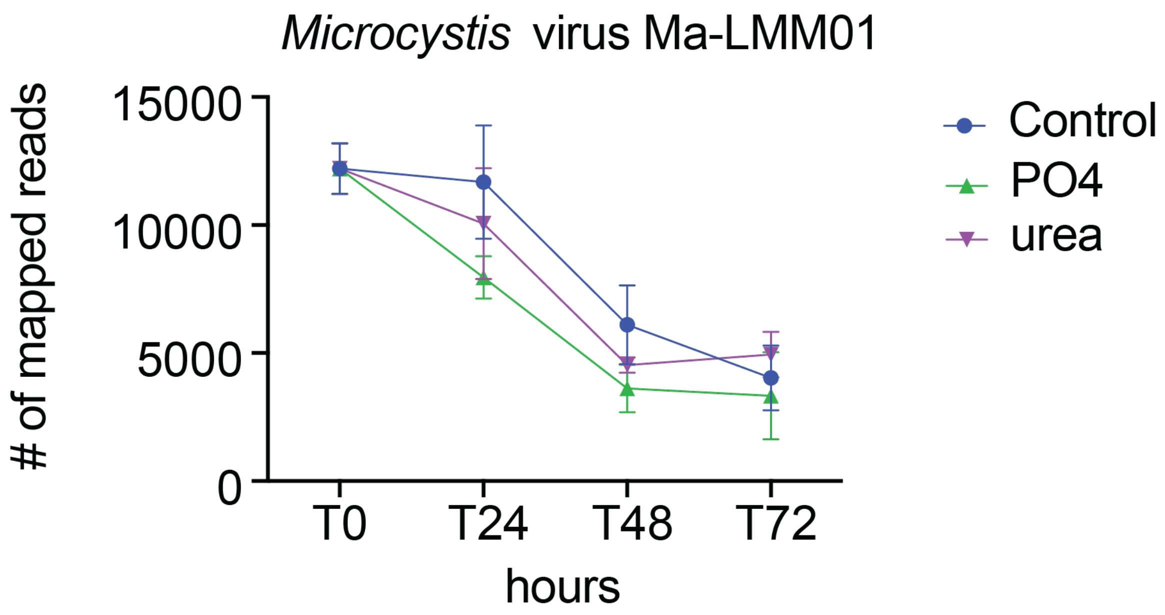

Figure S4. The expression of *Microcystis* phage is represented by reads mapped to the genome of *Microcystis* virus Ma-LMM01 during the mesocosm experiments.

Table S1. Nutrient dosing and timing for mesocosm experiments. Nutrients were added to all replicates in a treatment.

Time point	Treatment	Nutrient added	Amount added
T0	Urea	CH_4N_2O	50 μM N
	PO_4	$Na_2HPO_4 \cdot 12H_2O$	5 μM P
T24	Urea	CH_4N_2O	100 μM N
	PO_4	$Na_2HPO_4 \cdot 12H_2O$	10 μM P
T48	Urea	CH_4N_2O	150 μM N
	PO_4	$Na_2HPO_4 \cdot 12H_2O$	15 μM P

Table S2. Sample information for the metatranscriptomes generated in this study. NA indicates data was not relevant for that sample.

Treatment	Time point	Replicate	Date	Sample collection time	Reads	Nutrient addition time	Sample collection after nutrient addition
Control	T0	R1	5/17/21	2:36:00 PM	86,529,480	NA	3:33:00 PM
	T0	R2	5/17/21	2:34:00 PM	61,888,920	NA	3:31:00 PM
	T0	R3	5/17/21	2:35:00 PM	69,954,538	NA	3:32:00 PM
	T24	R1	5/18/21	1:18:00 PM	64,556,288	NA	2:18:00 PM
	T24	R2	5/18/21	1:15:00 PM	77,941,920	NA	2:26:00 PM
	T24	R3	5/18/21	1:17:00 PM	87,701,404	NA	2:19:00 PM
	T48	R1	5/19/21	1:29:00 PM	68,189,066	NA	2:45:00 PM
	T48	R2	5/19/21	1:26:00 PM	77,792,766	NA	2:47:00 PM
	T48	R3	5/19/21	1:28:00 PM	83,555,384	NA	2:46:00 PM
	T72	R1	5/20/21	11:45:00 AM	73,088,416	NA	NA
	T72	R2	5/20/21	11:42:00 AM	78,900,984	NA	NA
	T72	R3	5/20/21	11:44:00 AM	114,173,676	NA	NA
PO4	T24	R1	5/18/21	12:51:00 PM	74,690,868	1:29:00 PM	2:14:00 PM
	T24	R2	5/18/21	12:52:00 PM	62,229,040	1:30:00 PM	2:15:00 PM
	T24	R3	5/18/21	12:53:00 PM	63,374,972	1:31:00 PM	2:16:00 PM
	T48	R1	5/19/21	1:15:00 PM	79,221,342	1:48:00 PM	2:31:00 PM
	T48	R2	5/19/21	1:18:00 PM	98,081,498	1:49:00 PM	2:32:00 PM
	T48	R3	5/19/21	1:19:00 PM	76,098,100	1:50:00 PM	2:33:00 PM
	T72	R1	5/20/21	12:05:00 PM	70,121,534	NA	NA
	T72	R2	5/20/21	12:06:00 PM	70,126,632	NA	NA
	T72	R3	5/20/21	12:07:00 PM	93,134,922	NA	NA
Urea	T24	R1	5/18/21	1:17:00 PM	75,106,332	1:31:00 PM	2:15:00 PM
	T24	R2	5/18/21	1:15:00 PM	85,577,302	1:29:00 PM	2:14:00 PM
	T24	R3	5/18/21	1:18:00 PM	71,109,500	1:32:00 PM	2:16:00 PM
	T48	R1	5/19/21	1:35:00 PM	60,438,366	1:50:00 PM	2:35:00 PM
	T48	R2	5/19/21	1:37:00 PM	64,441,500	1:51:00 PM	2:36:00 PM
	T48	R3	5/19/21	1:33:00 PM	75,082,846	1:49:00 PM	2:34:00 PM
	T72	R1	5/20/21	11:57:00 AM	84,145,490	NA	NA
	T72	R2	5/20/21	11:56:00 AM	60,527,848	NA	NA
	T72	R3	5/20/21	11:58:00 AM	60,371,566	NA	NA
CR	NA	NA	5/17/22	2:05:00 PM	83,583,814	NA	NA
CR	NA	NA	5/18/22	1:14:00 PM	80,285,516	NA	NA
CR	NA	NA	5/19/22	2:30:00 PM	77,832,620	NA	NA
CR	NA	NA	5/20/22	12:08:00 PM	134,286,712	NA	NA

Table S3. Information for metagenomes generated from samples collected on Lake O in 2020 and 2021.

	Station	Month	Date sampled	Time	Reads
1	S308	June			83,624,248
2	S308	July	7/8/20	8:05:00 AM	68,014,022
3	S308	August	8/5/20	8:15:00 AM	75,783,650
4	L007	June	6/3/20	12:19:00 PM	59,251,456
5	LZ25A	July	7/8/20	9:40:00 AM	90,309,542
6	LZ25A	August	8/5/20	9:40:00 AM	65,531,768
7	L005	June	6/2/20	12:35:00 PM	72,856,672
8	L005	July	7/7/20	11:45:00 AM	80,112,150
9	L005	August	8/4/20	11:58:00 AM	61,965,342
10	LZ40	June	6/3/20	9:52:00 AM	74,376,858
11	LZ40	July	7/8/20	12:33:00 PM	73,875,040
12	LZ40	August	8/5/20	12:37:00 PM	70,989,040
13	KissR0.0	June	6/2/20	8:25:00 AM	73,239,768
14	KissR0.0	July	7/7/20	7:59:00 AM	110,486,900
15	KissR0.0	August	8/4/20	8:10:00 AM	85,856,550
16	Polesout	June	6/2/20	2:00:00 PM	84,252,642
17	Polesout	July	7/7/20	12:55:00 PM	87,841,092
18	Polesout	August	8/4/20	1:31:00 PM	77,846,440
19	S308	February	2/10/21	7:43:00 AM	76,171,106
20	S308	March	3/10/21	7:00:00 AM	54,499,994
21	S308	May	5/5/21	7:45:00 AM	115,773,192
22	CLV01A	July	7/7/21	1:15:00 PM	50,888,176
23	LZ25A	February	2/10/21	9:44:00 AM	29,293,698
24	LZ25A	March	3/11/21	11:40:00 AM	77,333,100
25	LZ25A	May	5/5/21	9:52:00 AM	143,673,530
26	LZ25A	July	7/7/21	9:12:00 AM	79,815,290
27	L005	February	2/9/21	2:00:00 PM	55,689,522
28	L005	March	3/10/21	11:14:00 AM	89,341,440
29	L005	May	5/4/21	12:02:00 PM	97,168,364
30	L005	July	7/8/21	11:32:00 AM	110,229,034

Table S3. cont'd

	Station	Month	Date sampled	Time	Reads
31	LZ40	February	2/10/21	1:52:00 PM	84,025,754
32	LZ40	March	3/11/21	8:30:00 AM	131,479,998
33	LZ40	May	5/5/21	12:46:00 PM	165,850,528
34	LZ40	July	7/7/21	12:36:00 PM	49,210,746
35	Polesout	February	2/9/21	1:24:00 PM	34,638,096
36	Polesout	March	3/10/21	12:50:00 PM	72,692,752
37	Polesout	May	5/4/21	1:27:00 PM	74,407,248
38	Polesout	July	7/8/21	12:52:00 PM	95,281,868
39	KissR0.0	February	2/9/21	8:56:00 AM	43,166,464
40	KissR0.0	March	3/10/21	7:06:00 AM	67,104,238
41	KissR0.0	May	5/4/21	7:40:00 AM	53,067,822
42	KissR0.0	July	7/8/21	7:37:00 AM	76,088,218
